# Mitochondrial haplogroup H is related to CD4+ T cell recovery in HIV infected patients starting combination antiretroviral therapy

**DOI:** 10.1186/s12967-018-1717-y

**Published:** 2018-12-06

**Authors:** Luz M. Medrano, Mónica Gutiérrez-Rivas, Julià Blanco, Marcial García, María A. Jiménez-Sousa, Yolanda M. Pacheco, Marta Montero, José Antonio Iribarren, Enrique Bernal, Onofre Juan Martínez, José M. Benito, Norma Rallón, Salvador Resino, CoRIS and the HIV Biobank integrated in the Spanish AIDS Research Network Project RIS/EPICLIN 10_2015

**Affiliations:** 1 Unidad de Infección Viral e Inmunidad, Centro Nacional de Microbiología, Instituto de Salud Carlos III, Carretera Majadahonda-Pozuelo, Km 2.2, 28220 Majadahonda, Madrid Spain; 2 Institut de Recerca de la Sida IrsiCaixa-HIVACAT, Badalona, Barcelona, Spain; 3 Institut d’investigació en Ciènces de la Salut Germans Trias i Pujol, Universitat Autónoma de Barcelona, Badalona, Barcelona, Spain; 4 Instituto de Investigación Sanitaria-Fundación Jiménez Díaz, Universidad Autónoma de Madrid (IIS-FJD, UAM), Av. Reyes Católicos, 2, 28040 Madrid, Spain; 5 Hospital Universitario Rey Juan Carlos, Móstoles, Madrid Spain; 6 Laboratorio de Immunobiología, Instituto de Biomedicina de Sevilla (IBiS), Seville, Spain; 7 UGC Clinical Laboratories, Hospital Universitario Virgen del Rocío, Seville, Spain; 8 Hospital Universitario y Politécnico de La Fe, Valencia, Spain; 9 Hospital Universitario Donostia, San Sebastián, Spain; 10 Hospital General Universitario Reina Sofía, Murcia, Spain; 11 Hospital General Universitario Santa Lucía, Cartagena, Spain

**Keywords:** HIV, Mitochondria, Haplogroups, mtDNA, Immune reconstitution, cART

## Abstract

**Background:**

The mitochondrial DNA (mtDNA) seems to influence in a large number of diseases, including HIV infection. Moreover, there is a substantial inter-individual variability in the CD4+ recovery in HIV-infected patients on combination antiretroviral therapy (cART). Our study aimed to analyze the association between mtDNA haplogroups and CD4+ recovery in HIV-infected patients on cART.

**Methods:**

This is a retrospective study of 324 naïve cART patients with CD4+ < 200 cells/mm^3^, who were followed-up during 24 months after initiating cART. All patients had undetectable HIV viral load during the follow-up. Besides, we included 141 healthy controls. MtDNA genotyping was performed by using Sequenom’s MassARRAY platform. The primary outcome variable was the slope of CD4+ recovery. Patients were stratified into two groups by the median slope value of CD4+ (9.65 CD4+ cells/mm^3^/month). Logistic regression analyses were performed to calculate the odds of CD4+ recovery according to mtDNA haplogroups.

**Results:**

Our study included European HIV-infected patients within the *N* macro-cluster. The baseline values of CD4+ T-cells were similar between groups of patients stratified by the P50th of the slope of CD4+ T-cells recovery. Patients in the low CD4+ T-cells recovery group were older (p = 0.001), but this variable was included in the multivariate models. When we analyzed the frequencies of mtDNA haplogroups, no significant differences between HIV-infected individuals and healthy controls were found. We did not find any significant association between mtDNA haplogroups and the slope of CD4+ T-cells recovery by linear regression analysis. However, Patients carrying haplogroup H had a higher odds of having a better CD4+ recovery (> 9.65 CD4+ cells/mm^3^/month) than patients without haplogroup H (p = 0.032). The adjusted logistic regression showed that patients carrying haplogroup H had a higher likelihood of achieving a CD4+ recovery > 9.65 CD4+ cells/mm^3^/month [adjusted odds ratio (aOR) = 1.75 (95% CI = 1.04; 2.95); p = 0.035].

**Conclusions:**

European mitochondrial haplogroup H was associated with the improved CD4+ recovery in HIV-infected patients starting cART with CD4+ < 200 cells/mm^3^.

**Electronic supplementary material:**

The online version of this article (10.1186/s12967-018-1717-y) contains supplementary material, which is available to authorized users.

## Background

The human immunodeficiency virus (HIV) infects and destroys the CD4+ T cell, promoting a continuous loss of CD4+ T cells that leads to immunodeficiency, opportunistic diseases, and death [1, 2]. The cART reduces the plasma HIV-RNA to undetectable levels and restores immunologic function, decreasing clinical progression and prolonging life [3]. However, despite suppression of HIV replication, a fraction of cART-treated patients fail to reconstitute CD4+ T-cell numbers sufficiently [4].

Mitochondrial dysfunction is related to acquired immune deficiency syndrome (AIDS) progression, in which there is mitochondrial DNA (mtDNA) depletion, increased reactive oxygen species (ROS) formation, antioxidant enzyme deficiency, and increased oxidative damage in patients with accelerated AIDS disease [5]. Additionally, mitochondrial toxicity due to nucleoside reverse transcriptase inhibitors may contribute to severe side effects observed in HIV-infected individuals on combination antiretroviral therapy (cART) [5].

Variations in mtDNA sequence are associated with several disorders [6], including HIV infection [5]. The combinations of mtDNA polymorphisms define mitochondrial haplogroups, which have a well-defined phylogeny among human populations [7]. In European Caucasians (N macro-cluster), people stratify in 4 cluster or major-haplogroups (HV, U, JT, and IWX) and several main haplogroups (H, V, pre-V, J, T, U, W, X, I) [7]. In European HIV-infected patients, mtDNA haplogroup has been associated with viroimmunological parameters, metabolic alterations, and AIDS progression [5, 8–10]. To our knowledge, only a previous study of our group has reported data about European mtDNA haplogroups and immune recovery in a cohort of HIV-infected patients starting cART with CD4+ count < 350 cells/mm^3^ in a tertiary hospital in Madrid (Spain) [11]. In the current multicentric study, we analyzed the association between European haplogroups and CD4+ T-cell recovery in naïve HIV-infected patients starting cART with CD4+ count < 200 cells/mm^3^ collected throughout the Spanish national territory.

## Methods

### Study population

We performed a retrospective study in 324 European HIV-infected patients receiving cART and 141 healthy controls of age and sex similar to patients. The Institutional Ethics Committee approved the study in concordance with the Declaration of Helsinki. All subjects provided informed consent to participate in the study.

The Spanish HIV BioBank of the CoRIS and the AIDS Research Institute IrsiCaixa-HIVACAT, Institut de Recerca en Ciències de la Salut Germans Trias i Pujol (Barcelona, Spain) provided patients. Epidemiological and clinical data were collected from medical records. The inclusion criteria were: (1) naïve for cART at inclusion in the cohort; (2) plasma HIV-RNA > 200 copies/mL; (3) starting cART with CD4+ counts < 200 cells/µL; (4) complete viral suppression (plasma HIV-RNA < 50 copies/mL) for two years after starting cART; (5) regular follow up of CD4+ counts and plasma HIV-RNA for two years after starting cART; (6) Individuals who were within the European N macro-haplogroup.

### DNA genotyping

The Spanish National Genotyping Center (CeGen; http://www.cegen.org/) genotyped DNA samples by using the iPLEX^®^ Gold technology and Agena Bioscience’s MassARRAY platform (San Diego, CA, USA). We selected 14 mtDNA polymorphisms within N macro-cluster [7, 11], to stratify patients by major-haplogroups or cluster (HV, IWX, U, and JT) and haplogroups (H, V, pre-V, J, T, I, W and X) (see Additional file 1).

### Outcome variables

The primary outcome variable was the slope of CD4+ T-cells (summary measure) during 2 years of follow-up. Patients were stratified in two groups by the median value of CD4+ T-cells slope in the whole population of patients (9.65 CD4+ cells/mm^3^/month): high CD4+ recovery (≥ 9.65 CD4+ cells/mm^3^/month) and low CD4+ recovery (< 9.65 CD4+ cells/mm^3^/month).

### Statistical analysis

Statistical analysis was performed with SPSS 22.0 software (SPSS INC, Chicago, IL, USA). All tests were two-tailed with p-values ≤ 0.05 considered significant. Categorical data and proportions were analyzed by using Chi squared test or Fisher´s exact test. Mann–Whitney U test was used to compare data between independent groups when the variables were continuous.

Generalized Linear Models (GLM) with a gamma distribution (log-link) were used to evaluate the differences in the slope of CD4+ T-cells recovery (continuous variable). Logistic regression models were used to calculate the odds of higher or lower CD4+ recovery according to mtDNA haplogroups, one for each haplogroup evaluated. The multivariate regression tests were adjusted by the main clinical characteristics at baseline: gender, age, length of HIV-infection, baseline CD4+ cells/mm^3^, HIV transmission route, hepatitis C and hepatitis B coinfection, and type of cART regimen.

## Results

### Characteristics of the study population

Our study included European HIV-infected patients within the N macro-cluster [7]. From the total Spanish AIDS Research Network (CoRIS) and AIDS Research Institute IrsiCaixa-HIVACAT Cohorts (n = 6160 HIV-patients) we only selected the HIV-patients meeting the inclusion criteria described in Methods section (n = 418). Lastly, 94 individuals were excluded from the analysis either because genotyping was not valid (n = 13) or because the haplogroup did not belong to the N-cluster or to a European haplogroup (n = 81). Thus, the final analysis included 324 patients (see Additional file 2). The baseline characteristics of patients stratified by the P50th of the slope of CD4+ T-cells recovery are shown in Table 1. Patients in the low CD4+ T-cells recovery group were older (p = 0.001), but this variable was included in the multivariate models. Moreover, the baseline values of CD4+ T-cells were similar between groups, but after 2 years of successful cART, the values CD4+ T-cells were higher in patients with high CD4+ recovery than low CD4+ recovery [513 (418.4; 636.5) vs. 274 (176.7; 365.6); p < 0.001].

**Table 1:** Baseline clinical and epidemiological characteristics of HIV infected patients

Characteristics	All patients	HIV groups		
		Low CD4+ recovery	High CD4+ recovery	*p*-value
No.	324	162	162	
Male	264 (81.5%)	138 (85.2%)	126 (77.8%)	0.086
Age (years)	41.0 (34.7; 49.2)	43 (36.4; 51.3)	39.9 (33.8; 46.5)	*0.001*
Time since HIV diagnosis (years)	1 (1.0; 1.0)	1 (1.0; 2.0)	1 (1.0; 1.0)	0.117
CD4+ cell count at baseline (cells/μL)	105 (41; 159)	93.3 (37.5;147)	114.5 (41.5;165)	0.230
Hepatitis C infection	29 (8.9%)	16 (9.9%)	13 (8.0%)	0.559
Hepatitis B infection	14 (4.3%)	8 (4.9%)	6 (3.7%)	0.585
cART regimen				
PI-based	103 (31.8%)	45 (28.0%)	58 (35.8%)	0.469
NNRTI-based	165 (51.1%)	88 (54.7%)	77 (47.5%)	
PI+ NNRTI-based	38 (11.8%)	20 (12.4%)	18 (11.1%)	
Others	17 (5.3%)	8 (5.0%)	9 (5.6%)	
HIV transmission route				
IDU	51 (17.1%)	30 (20.8%)	21 (13.4%)	0.219
Homosexual transmission	152 (50.8%)	68 (47.2%)	84 (54.2%)	
Heterosexual transmission	96 (32.1%)	46 (31.9%)	50 (32.3%)	

*IDU* intravenous drug users, *HIV* human immunodeficiency virus, *cART* combination antiretroviral therapy, *PI* HIV protease inhibitor, *NNRTI* non-nucleoside analogue HIV reverse transcriptase inhibitor

Statistical: Values were expressed as absolute number (percentage) and median (percentile 25; percentile 75). Significant differences are shown in bold. p-values were calculated by Chi square and Mann–Whitney tests

### Characteristics of mtDNA haplogroups

**Fig. 1:**
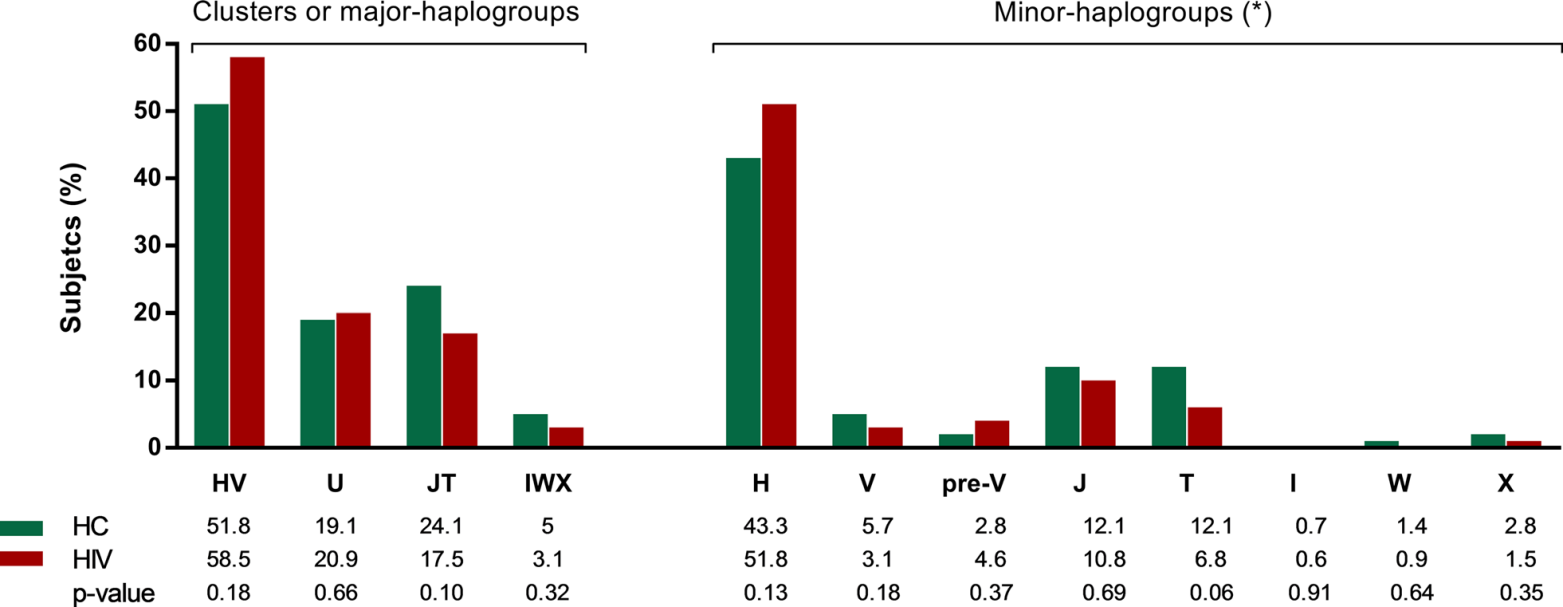
Frequencies of mtDNA haplogroups in HIV infected patients and healthy controls. Statistical: p-values were calculated by Chi square test. *The percentages of mtDNA haplogroups, for both HIV and HC groups, do not add to 100% because the cluster U was not stratified in minor-haplogroups. *HIV* human immunodeficiency virus, *HC* healthy controls

When we analyzed the frequencies of mtDNA haplogroups, no significant differences between HIV-infected individuals and healthy controls were found (Fig. 1). Note that healthy controls were similar to HIV-infected patients regarding gender and age. Moreover, the cluster IWX and minor haplogroups V, pre-V, I, X, and W were discarded for the genetic association study because these mtDNA haplogroups had low frequencies (< 5%) in HIV infected patients (Fig. 1). Thus, the genetic association tests were performed on the clusters HV, U, and JT; and on the haplogroups H, J, and T.

### mtDNA haplogroups and CD4+ T-cell recovery

**Fig. 2:**
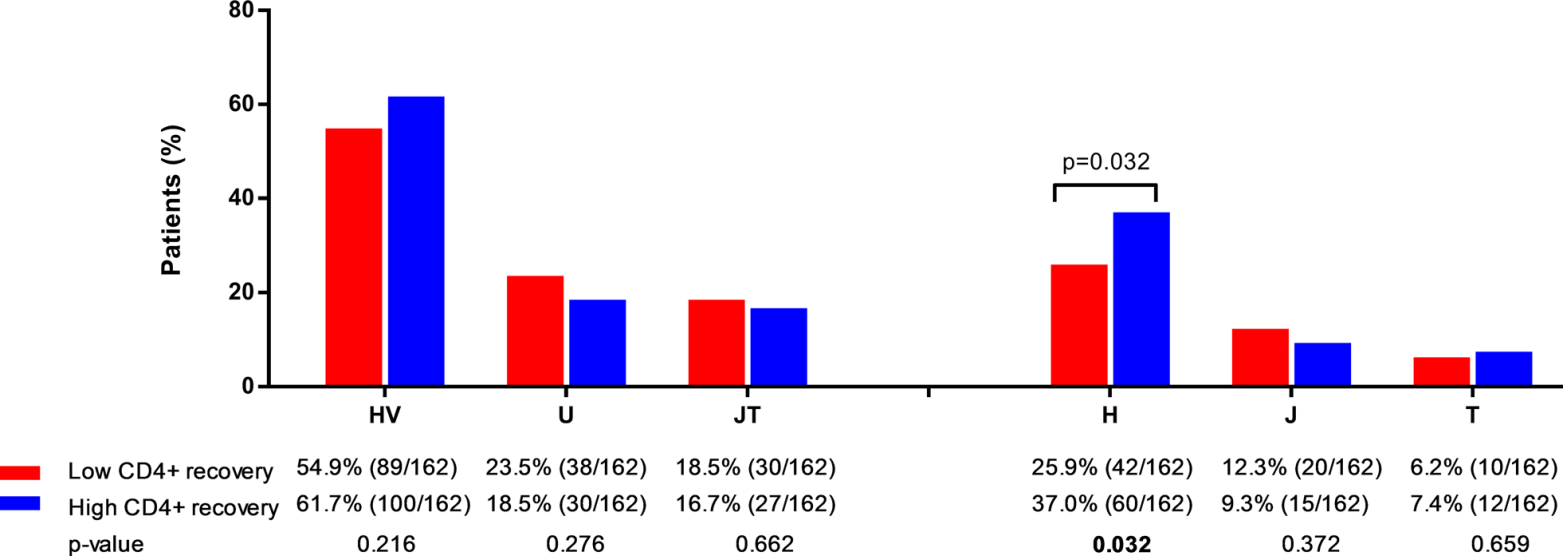
Percentage of HIV-infected patients according to mtDNA haplogroups and CD4+ T cell recovery during the follow-up. Statistical: p-values were calculated by Chi squared test or Fisher’s exact test

**Fig. 3:**
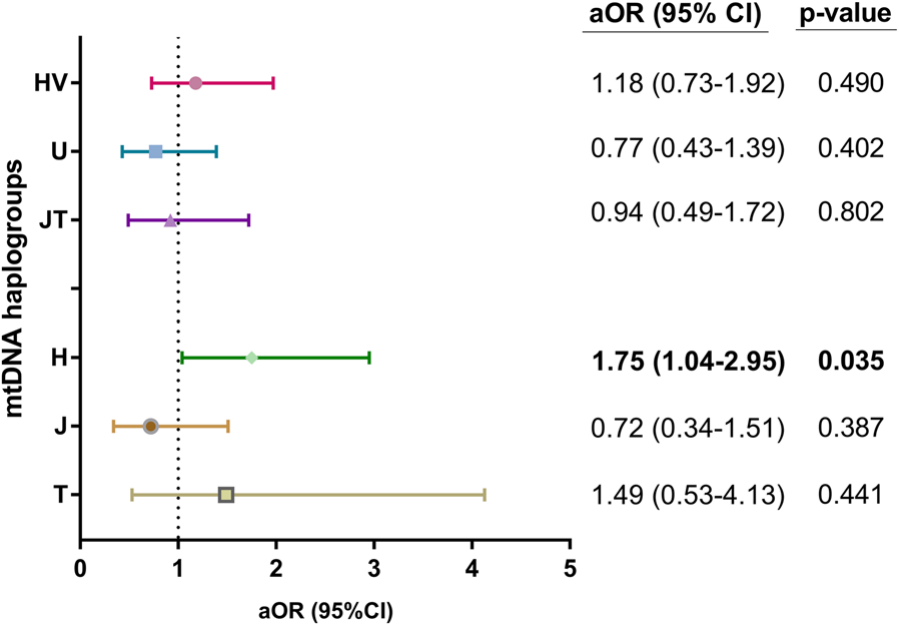
Summary of the association between mtDNA haplogroups and CD4+ T cell recovery in HIV-infected patients who started combination antiretroviral therapy. Statistical: Logistic regression models were used to calculate the odds of higher or lower CD4+ recovery according to mtDNA haplogroups, one for each haplogroup evaluated. These regressions were adjusted by the most important clinical and epidemiological characteristics (gender, age, length of HIV-infection, baseline CD4+ cells/mm^3^, HIV transmission route, hepatitis C and hepatitis B coinfection, and cART regimen). *aOR* adjusted odds ratio, *95% CI* 95% of confidence interval

We did not find any significant association between mtDNA haplogroups and the slope of CD4+ T-cells recovery by linear regression analysis (Table 2). However, we found significant values when patients were stratified by the P50th of the slope of CD4+ T-cells recovery (Fig. 2). We found higher frequency of H haplogroup in patients with high CD4+ recovery (≥ 9.65 CD4+ cells/mm^3^/month) (p = 0.032). On the other hand, in multivariate analysis adjusted by the main clinical characteristics at baseline (see statistical analysis section), patients with haplogroup H had higher odds of having a better CD4+ T-cell recovery than patients without haplogroup H [adjusted odds ratio (aOR) = 1.75 (95% CI = 1.04; 2.95); p = 0.035]. No significant associations were found for the others haplogroups (Fig. 3; full description in Additional file 3).

**Table 2:** Summary of the slopes of CD4+ T-cells recovery (cells/mm^3^/month) for each mtDNA haplogroup and the relationship between them in HIV-infected patients who started combined antiretroviral therapy

Mt DNA haplogroups	Unadjusted analysis*			Adjusted analysis**	
	No	Yes	*p*-value	aAMR (95% CI)	*p*-value
Clusters or major haplogroups					
HV	9.1 (5.5; 14)	10 (6.4; 14.7)	0.288	0.98 (0.86; 1.13)	0.839
U	9.8 (6.2; 14.1)	9.1 (5.1; 13.9)	0.321	0.91 (0.77; 1.08)	0.300
JT	9.8 (6.2; 14)	9.2 (5.6; 13.6)	0.765	1.12 (0.94; 1.33)	0.205
Haplogroups					
H	9.1 (5.6; 13.7)	10.2 (6.9; 14.1)	0.075	1.04 (0.91; 1.21)	0.544
J	9.8 (6.2; 14)	8.8 (5.2; 12.2)	0.522	1.07 (0.86; 1.32)	0.537
T	9.6 (6.1; 14)	9.7 (6.8; 14.2)	0.735	1.17 (0.90; 1.51)	0.225

*HIV* human immunodeficiency virus, *aAMR* adjusted arithmetic mean ratio, *CI* confidence interval

Statistical: * Values were expressed as median (cells/mm^3^/month) (percentile 25; percentile 75). p-values were calculated by Mann–Whitney tests. ** Values were expressed as adjusted arithmetic mean ratio (aAMR) and 95% of confidence interval (95% CI) of the slopes of CD4+ T-cells recovery (cells/mm^3^/month). p-values were calculated by Generalized Linear Models test with a normal distribution (log-link). These regressions were adjusted by the most important clinical and epidemiological characteristics (gender, age, length of HIV-infection, baseline CD4+ cells/mm^3^, HIV transmission route related to IDU, hepatitis C and hepatitis B coinfection, and type of cART regimen)

## Discussion

In this study, haplogroup H was related to better CD4+ T-cells counts recovery in HIV-infected patients starting cART with low CD4 counts and followed during the first 24 months after initiating cART. This association was found both in the univariate and in the multivariate analysis adjusted by the most important baseline characteristics. However, we did not find any association when the continuous outcome measure (slope of CD4+ T-cells recovery) was used, possibly because there is no linear relationship between the analyzed variables.

The data found in this study confirm the positive influence of European haplogroup H on CD4+ T-cells count recovery in cART-treated patients, which was previously described by our group in a different cohort [11]. In addition to the influence of haplogroup H, in this previous article of Guzmán-Fulgencio et al. [11], we also found a worse CD4+ recovery in patients with haplogroup J and T. However, we did not observe any significant association for haplogroups J and T in the current study. The small sample size could explain this discrepancy in these haplogroups, which may have impaired the ability to detect less robust associations. However, we must also consider other factors. On the one hand, the article of Guzmán-Fulgencio et al. [11] studied a cohort of HIV-infected patients provided by a tertiary referral hospital center (Spain), with baseline CD4+ values < 350 cells/mm^3^, and followed during at least 24 months. On the other hand, we now analyzed a cohort of HIV-infected patients provided by a large number of hospitals spread throughout Spain (a sample more representative of Spanish population), with baseline CD4+ values < 200 cells/mm^3^ (a more restricted criteria), and followed during the first 24 months after starting cART (the same follow-up in all patients). Moreover, the type of statistical analysis applied was different in our previous study. In the article of Guzmán-Fulgencio et al. [11], a survival analysis was performed with CD4+ > 500 cells/mm^3^ as the primary outcome, whereas in the present study, we calculated the slope of CD4+ T-cells count for each patient and compared groups by logistic regression analysis.

Moreover, mtDNA haplogroups in other cohort have also been related to CD4+ T-cells recovery in HIV-infected patients who started cART. Grady et al. found that African L2 haplogroup was associated with decreased odds of CD4+ T-cells recovery after cART (CD4+ count change of ≥ 100 cells/mm^3^ and median CD4+ T-cell increases at 48 weeks of follow-up) in the AIDS Clinical Trials Group study 384 [12]. However, they did not find any significant association with European mitochondrial haplogroups in non-Hispanic white participants.

In the present study, the age of HIV-infected patients with worse CD4+ T-cells recovery was higher than patients with better CD4+ T-cells recovery, which could have had a negative impact since a poorer CD4+ T-cell counts recovery has been found in older HIV-infected patients starting cART [13]. However, we think that only 3 years of difference between groups is not relevant in adults of 35–45 years. In fact, we included the age in the multivariate analysis, and the significant association between haplogroup H and CD4+ T-cells recovery was maintained.

Variants in mtDNA which are not silent may have an essential role in adaptation to environmental conditions, by modulating specific mitochondrial functions [14]. Thus, the differences found in mtDNA haplogroups distribution among the different groups of patients in our study may be because haplogroup H is related to higher activity in the electron transport chain, producing higher levels of ATP and ROS than other haplogroups [15–17], increasing the immune response against HIV infection [9]. Furthermore, ROS production may lead to an up-regulation of antioxidant defenses without causing severe immune damage [18], contributing to proper immune function, ensuring control of HIV replication and, in turn, decreasing oxidative stress and apoptosis [19, 20]. Additionally, a higher degree of energetic efficiency could have a substantial impact on HIV infection since the efficiency of the metabolism regulate T-cell function and susceptibility to infection [21]. The functional role of mtDNA haplogroups is controversial, but it is possible that the effects of mitochondrial haplogroups may emerge under special conditions such as CD4+ T-cells recovery in HIV-infected patients starting cART. However, we could not perform functional experiments to determinate the energetic efficiency in isolated mitochondria of T-cells from patients.

Nowadays or in the future, our results may have an impact on clinical practice in those cases where cART starts with very low CD4+ T-cells. cART should begin in all HIV-infected patients, regardless of the CD4+ T-cells count in order to decrease the risk of HIV transmission and prevent AIDS-related illness [22]. However, late presentation for HIV care is a significant and persistent issue throughout the world [23–25], including developed countries with health systems with good access to health services [24]. Late presenters have delayed initiation of cART, CD4 values below 350 cells/mm^3^ and below 200 cells/mm^3^ in many cases [26], and higher risk of AIDS progression and death [27]. The initiation of cART with very low CD4+ T-cell counts is consistently associated with poorer outcomes of cART [28]. Thus, our data could provide information to improve the management of HIV-infected patients with poorer prognosis of CD4+ T-cells recovery.

## Study limitations

Our study has some limitations that we should take into account to make a correct interpretation of the results. Firstly, this is a retrospective study and, therefore, the case record is selected a priori from patients surviving long enough to yield sufficient follow-up (at least 24 months after cART). Secondly, the sample size is limited, particularly in some haplogroups, which may have impaired the ability to detect less robust associations. Thirdly, although our results suggest that some variants in mtDNA may influence CD4+ T-cell recovery, it would be necessary to perform functional ROS and ATP measurement in the patients to provide additional confirmatory data. Fourthly, this study was carried out on Caucasian patients, and our conclusions are only truly applicable to this population.

## Conclusions

In conclusion, European haplogroup H was associated with the improved CD4+ recovery in HIV-infected patients starting cART with CD4+ < 200 cells/mm^3^. This finding could be useful to understand the host genetic factors involved in the immune recovery of cART-treated patients. Additionally, it also may help to improve the management of patients with a poorer prognosis of immune recovery.

## Additional files

**Additional file 1.** Summary of European mitochondrial DNA (mtDNA) haplogroups with their defining polymorphisms.

**Additional file 2.** Flow chart showing the sequential steps to select the HIV-patients included in the study.

**Additional file 3.** Summary of full multivariate model results for the regression between mitochondrial DNA (mtDNA) haplogroups and CD4+ T cell recovery in HIV-infected patients who started combination antiretroviral therapy.

## Abbreviations

HIV: human immunodeficiency virus
AIDS: acquired immune deficiency syndrome
mtDNA: mitochondrial DNA
cART: combination antiretroviral therapy
ROS: reactive oxygen species
