# Supplementary figures and images for: Mitochondrial haplogroup H is related to CD4+ T cell recovery in HIV infected patients starting combination antiretroviral therapy
Source: J Transl Med. 2018 Dec 6;16:343. doi: 10.1186/s12967-018-1717-y (PMC6282399; doi:10.1186/s12967-018-1717-y)

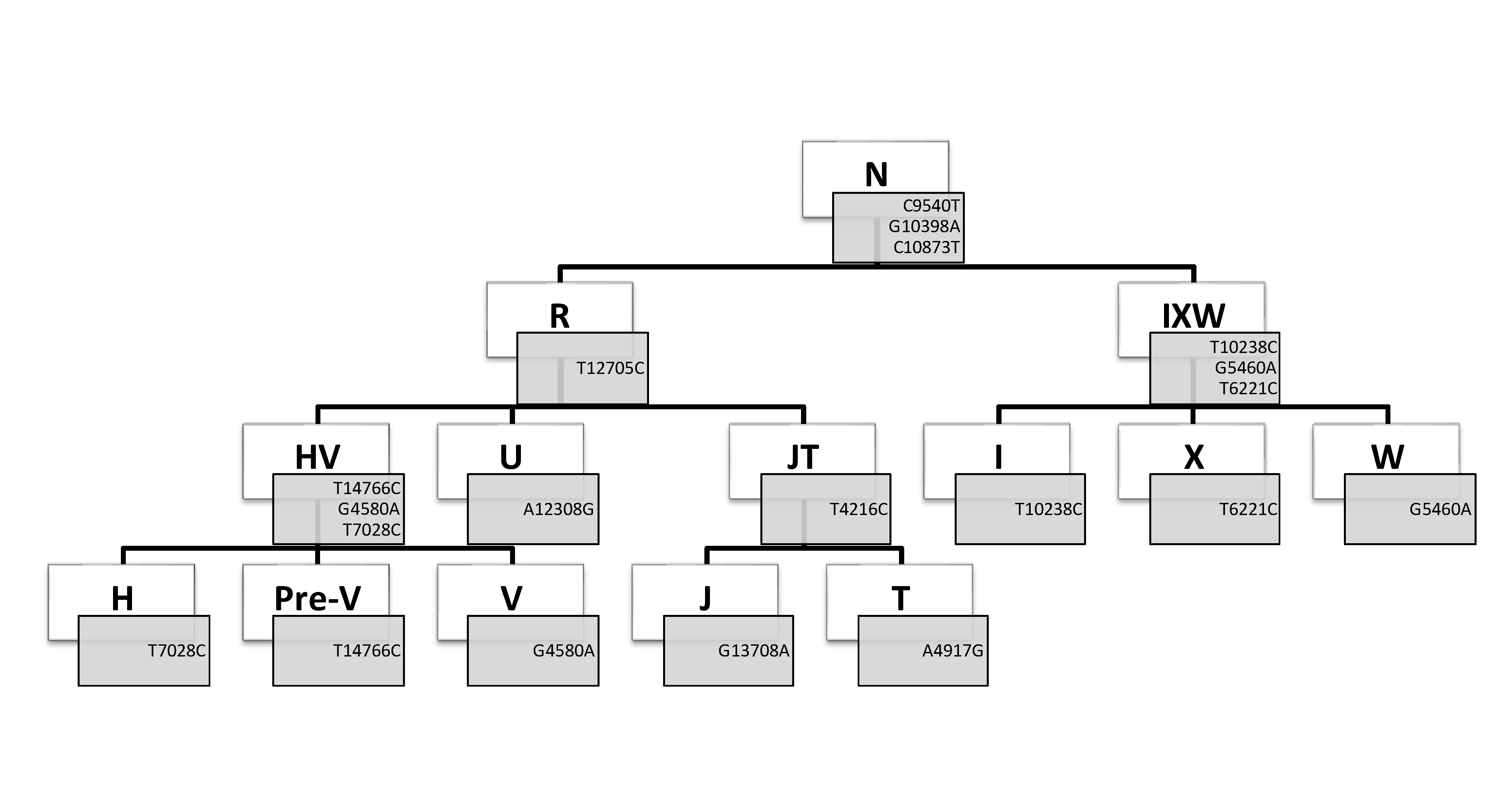

Supplement: Supplementary file 1 — Additional file 1. Summary of European mitochondrial DNA (mtDNA) haplogroups with their defining polymorphisms. [file 12967_2018_1717_MOESM1_ESM.tif]

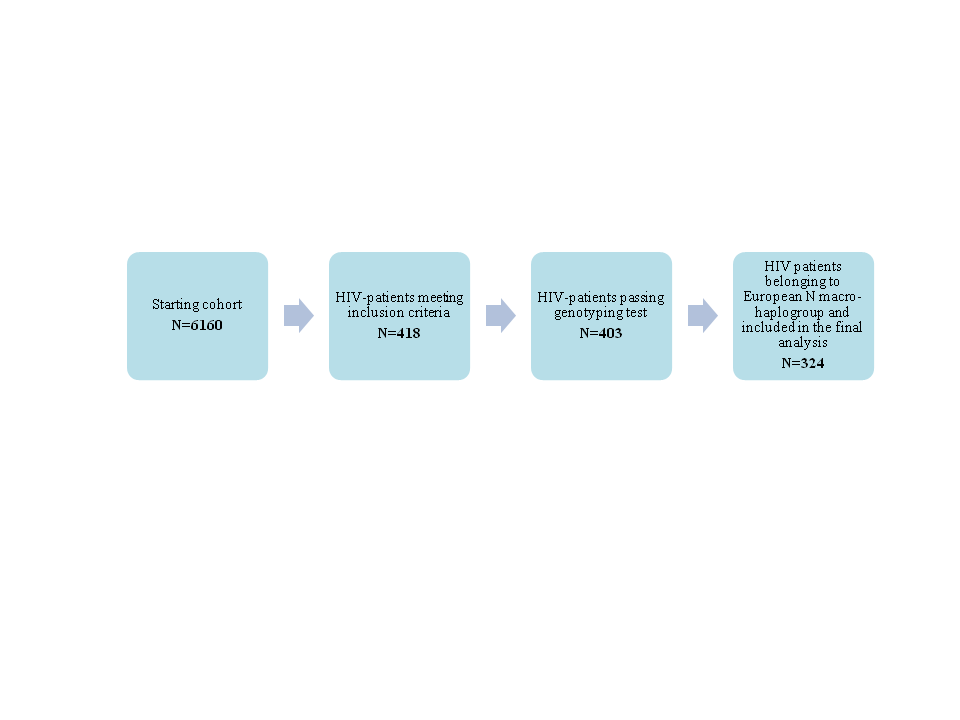

Supplement: Supplementary file 2 — Additional file 2. Flow chart showing the sequential steps to select the HIV-patients included in the study. [file 12967_2018_1717_MOESM2_ESM.tif]
