# Supplementary material for: Mitochondrial haplogroup H is related to CD4+ T cell recovery in HIV infected patients starting combination antiretroviral therapy
Source: J Transl Med. 2018 Dec 6;16:343. doi: 10.1186/s12967-018-1717-y (PMC6282399; doi:10.1186/s12967-018-1717-y)
Supplement: Supplementary file 3 — Additional file 3. Summary of full multivariate model results for the regression between mitochondrial DNA (mtDNA) haplogroups and CD4+ T cell recovery in HIV-infected patients who started combination antiretroviral therapy. [file 12967_2018_1717_MOESM3_ESM.docx]

# **Clusters or major-haplogroups**

| **Variables in the Equation** | | | | | | | | | |
| --- | --- | --- | --- | --- | --- | --- | --- | --- | --- |
|  | | B | S.E. | Wald | df | Sig. | Exp(B) | 95% C.I.for EXP(B) | |
|  |  |  |  |  |  |  |  | Lower | Upper |
| Step 1^a^ | HV | ,171 | ,248 | ,476 | 1 | ,490 | 1,186 | ,730 | 1,927 |
|  | HCV | -,074 | ,487 | ,023 | 1 | ,879 | ,929 | ,358 | 2,410 |
|  | HBV | -,393 | ,602 | ,426 | 1 | ,514 | ,675 | ,207 | 2,198 |
|  | Base_CD4 | ,005 | ,002 | 4,533 | 1 | ,033 | 1,005 | 1,000 | 1,009 |
|  | Age | -,038 | ,012 | 10,033 | 1 | ,002 | ,963 | ,940 | ,986 |
|  | Male | -,514 | ,327 | 2,479 | 1 | ,115 | ,598 | ,315 | 1,134 |
|  | Years_infection | -,012 | ,034 | ,130 | 1 | ,719 | ,988 | ,925 | 1,056 |
|  | PI_cART | -,367 | ,254 | 2,096 | 1 | ,148 | ,693 | ,421 | 1,139 |
|  | AIDS_diagnosis | ,685 | ,299 | 5,269 | 1 | ,022 | 1,985 | 1,105 | 3,563 |
|  | IDU | -,528 | ,400 | 1,744 | 1 | ,187 | ,590 | ,270 | 1,291 |
|  | Constant | 1,638 | ,685 | 5,720 | 1 | ,017 | 5,145 |  |  |
| a. Variable(s) entered on step 1: HCV, HBV, Base_CD4, Age, Male, Years_infection, PI_cART, AIDS_diagnosis, IDU. | | | | | | | | | |

| **Variables in the Equation** | | | | | | | | | |
| --- | --- | --- | --- | --- | --- | --- | --- | --- | --- |
|  | | B | S.E. | Wald | df | Sig. | Exp(B) | 95% C.I.for EXP(B) | |
|  |  |  |  |  |  |  |  | Lower | Upper |
| Step 1^a^ | U | -,251 | ,299 | ,704 | 1 | ,402 | ,778 | ,433 | 1,398 |
|  | HCV | -,105 | ,487 | ,047 | 1 | ,829 | ,900 | ,346 | 2,340 |
|  | HBV | -,374 | ,605 | ,382 | 1 | ,536 | ,688 | ,210 | 2,251 |
|  | Base_CD4 | ,005 | ,002 | 4,479 | 1 | ,034 | 1,005 | 1,000 | 1,009 |
|  | Age | -,038 | ,012 | 10,001 | 1 | ,002 | ,963 | ,940 | ,986 |
|  | Male | -,507 | ,326 | 2,410 | 1 | ,121 | ,603 | ,318 | 1,142 |
|  | Years_infection | -,014 | ,034 | ,176 | 1 | ,675 | ,986 | ,923 | 1,053 |
|  | PI_cART | -,377 | ,254 | 2,196 | 1 | ,138 | ,686 | ,417 | 1,129 |
|  | AIDS_diagnosis | ,680 | ,299 | 5,184 | 1 | ,023 | 1,975 | 1,099 | 3,546 |
|  | IDU | -,516 | ,399 | 1,671 | 1 | ,196 | ,597 | ,273 | 1,305 |
|  | Constant | 1,796 | ,673 | 7,122 | 1 | ,008 | 6,027 |  |  |
| a. Variable(s) entered on step 1: HCV, HBV, Base_CD4, Age, Male, Years_infection, PI_cART, AIDS_diagnosis, IDU. | | | | | | | | | |

| **Variables in the Equation** | | | | | | | | | |
| --- | --- | --- | --- | --- | --- | --- | --- | --- | --- |
|  | | B | S.E. | Wald | df | Sig. | Exp(B) | 95% C.I.for EXP(B) | |
|  |  |  |  |  |  |  |  | Lower | Upper |
| Step 1^a^ | JT | -,079 | ,317 | ,063 | 1 | ,802 | ,924 | ,496 | 1,720 |
|  | HCV | -,063 | ,489 | ,017 | 1 | ,898 | ,939 | ,360 | 2,447 |
|  | HBV | -,415 | ,601 | ,478 | 1 | ,489 | ,660 | ,203 | 2,143 |
|  | Base_CD4 | ,005 | ,002 | 4,696 | 1 | ,030 | 1,005 | 1,000 | 1,009 |
|  | Age | -,038 | ,012 | 10,044 | 1 | ,002 | ,963 | ,940 | ,986 |
|  | Male | -,533 | ,326 | 2,678 | 1 | ,102 | ,587 | ,310 | 1,111 |
|  | Years_infection | -,013 | ,034 | ,150 | 1 | ,699 | ,987 | ,924 | 1,055 |
|  | PI_cART | -,355 | ,253 | 1,961 | 1 | ,161 | ,701 | ,427 | 1,152 |
|  | AIDS_diagnosis | ,697 | ,298 | 5,469 | 1 | ,019 | 2,007 | 1,119 | 3,598 |
|  | IDU | -,517 | ,399 | 1,677 | 1 | ,195 | ,596 | ,273 | 1,304 |
|  | Constant | 1,747 | ,670 | 6,794 | 1 | ,009 | 5,735 |  |  |
| a. Variable(s) entered on step 1: HCV, HBV, Base_CD4, Age, Male, Years_infection, PI_cART, AIDS_diagnosis, IDU. | | | | | | | | | |

# **Minor-haplogroups**

| **Variables in the Equation** | | | | | | | | | |
| --- | --- | --- | --- | --- | --- | --- | --- | --- | --- |
|  | | B | S.E. | Wald | df | Sig. | Exp(B) | 95% C.I.for EXP(B) | |
|  |  |  |  |  |  |  |  | Lower | Upper |
| Step 1^a^ | H | ,562 | ,266 | 4,456 | 1 | ,035 | 1,755 | 1,041 | 2,958 |
|  | HCV | -,022 | ,492 | ,002 | 1 | ,964 | ,978 | ,373 | 2,568 |
|  | HBV | -,403 | ,603 | ,446 | 1 | ,504 | ,669 | ,205 | 2,180 |
|  | Base_CD4 | ,005 | ,002 | 4,418 | 1 | ,036 | 1,005 | 1,000 | 1,009 |
|  | Age | -,039 | ,012 | 10,303 | 1 | ,001 | ,962 | ,939 | ,985 |
|  | Male | -,503 | ,329 | 2,346 | 1 | ,126 | ,605 | ,318 | 1,151 |
|  | Years_infection | -,010 | ,034 | ,082 | 1 | ,774 | ,990 | ,926 | 1,059 |
|  | PI_cART | -,370 | ,256 | 2,086 | 1 | ,149 | ,691 | ,418 | 1,141 |
|  | AIDS_diagnosis | ,703 | ,300 | 5,498 | 1 | ,019 | 2,020 | 1,122 | 3,636 |
|  | IDU | -,610 | ,404 | 2,276 | 1 | ,131 | ,543 | ,246 | 1,200 |
|  | Constant | 1,587 | ,680 | 5,444 | 1 | ,020 | 4,890 |  |  |
| a. Variable(s) entered on step 1: HCV, HBV, Base_CD4, Age, Male, Years_infection, PI_cART, AIDS_diagnosis, IDU. | | | | | | | | | |

| **Variables in the Equation** | | | | | | | | | |
| --- | --- | --- | --- | --- | --- | --- | --- | --- | --- |
|  | | B | S.E. | Wald | df | Sig. | Exp(B) | 95% C.I.for EXP(B) | |
|  |  |  |  |  |  |  |  | Lower | Upper |
| Step 1^a^ | J | -,328 | ,379 | ,748 | 1 | ,387 | ,720 | ,343 | 1,515 |
|  | HCV | -,083 | ,486 | ,029 | 1 | ,865 | ,921 | ,355 | 2,388 |
|  | HBV | -,455 | ,603 | ,569 | 1 | ,451 | ,635 | ,195 | 2,068 |
|  | Base_CD4 | ,005 | ,002 | 4,796 | 1 | ,029 | 1,005 | 1,001 | 1,009 |
|  | Age | -,038 | ,012 | 9,869 | 1 | ,002 | ,963 | ,940 | ,986 |
|  | Male | -,523 | ,327 | 2,561 | 1 | ,110 | ,593 | ,313 | 1,125 |
|  | Years_infection | -,012 | ,034 | ,131 | 1 | ,717 | ,988 | ,925 | 1,055 |
|  | PI_cART | -,359 | ,253 | 2,003 | 1 | ,157 | ,699 | ,425 | 1,148 |
|  | AIDS_diagnosis | ,691 | ,298 | 5,365 | 1 | ,021 | 1,995 | 1,112 | 3,578 |
|  | IDU | -,522 | ,399 | 1,715 | 1 | ,190 | ,593 | ,271 | 1,296 |
|  | Constant | 1,750 | ,670 | 6,815 | 1 | ,009 | 5,755 |  |  |
| a. Variable(s) entered on step 1: HCV, HBV, Base_CD4, Age, Male, Years_infection, PI_cART, AIDS_diagnosis, IDU. | | | | | | | | | |

| **Variables in the Equation** | | | | | | | | | |
| --- | --- | --- | --- | --- | --- | --- | --- | --- | --- |
|  | | B | S.E. | Wald | df | Sig. | Exp(B) | 95% C.I.for EXP(B) | |
|  |  |  |  |  |  |  |  | Lower | Upper |
| Step 1^a^ | T | ,401 | ,520 | ,593 | 1 | ,441 | 1,493 | ,538 | 4,139 |
|  | HCV | -,147 | ,495 | ,088 | 1 | ,767 | ,863 | ,327 | 2,279 |
|  | HBV | -,466 | ,608 | ,588 | 1 | ,443 | ,627 | ,191 | 2,065 |
|  | Base_CD4 | ,005 | ,002 | 4,851 | 1 | ,028 | 1,005 | 1,001 | 1,009 |
|  | Age | -,038 | ,012 | 10,135 | 1 | ,001 | ,962 | ,940 | ,985 |
|  | Male | -,511 | ,326 | 2,453 | 1 | ,117 | ,600 | ,316 | 1,137 |
|  | Years_infection | -,017 | ,034 | ,238 | 1 | ,625 | ,984 | ,921 | 1,051 |
|  | PI_cART | -,379 | ,254 | 2,219 | 1 | ,136 | ,684 | ,416 | 1,127 |
|  | AIDS_diagnosis | ,701 | ,298 | 5,543 | 1 | ,019 | 2,015 | 1,125 | 3,611 |
|  | IDU | -,494 | ,399 | 1,531 | 1 | ,216 | ,610 | ,279 | 1,335 |
|  | Constant | 1,713 | ,668 | 6,573 | 1 | ,010 | 5,548 |  |  |
| a. Variable(s) entered on step 1: HCV, HBV, Base_CD4, Age, Male, Years_infection, PI_cART, AIDS_diagnosis, IDU. | | | | | | | | | |
